# The Reproductive Toxicity of Mequindox in a Two-Generation Study in Wistar Rats

**DOI:** 10.3389/fphar.2018.00870

**Published:** 2018-08-17

**Authors:** Qianying Liu, Zhixin Lei, Qin Wu, Ihsan Awais, Muhammad A. B. Shabbir, Saeed Ahmed, Zainab Fatima, Xu Wang, Yuanhu Pan, Shuyu Xie, Zonghui Yuan

**Affiliations:** ^1^National Reference Laboratory of Veterinary Drug Residues (HZAU) and MAO Key Laboratory for Detection of Veterinary Drug Residues, Huazhong Agricultural University, Wuhan, China; ^2^MOA Laboratory for Risk Assessment of Quality and Safety of Livestock and Poultry Products, Huazhong Agricultural University, Wuhan, China; ^3^Hubei Collaborative Innovation Center for Animal Nutrition and Feed Safety, Wuhan, China

**Keywords:** reproductive toxicity, teratogenicity, mequindox, Wistar rats, developmental toxicity

## Abstract

Mequindox (MEQ), belonging to quinoxaline-di-*N*-oxides (QdNOs), has been extensively used as a synthetic antibacterial agent. To evaluate the reproductive toxicity of MEQ, different concentrations of MEQ were administered to Wistar rats by feeding diets containing 0, 25, 55, 110, and 275 mg/kg, respectively. Each group consisting of 25 males and 25 females (F_0_) was treated with different concentrations of MEQ for 12-week period time, prior to mating and during mating, gestation, parturition and lactation. At weaning, 25 males and 25 females of F_1_ generation weanlings per group were randomly selected as parents for the F_2_ generation. Selected F_1_ weanlings were exposed to the same diet and treatment as their parents. The number of live litter and indexes of mating and fertility were significantly decreased in the F1 and F2 generation at 110 and 275 mg/kg groups. Significant decrease in pup vitality during lactation was observed in F1 litter at 275 mg/kg group, in F2 litter at 55, 110, and 275 mg/kg groups. A downward trend in the body weights was observed in F_1_ pups at 55, 110, and 275 mg/kg MEQ groups, and in F_2_ pups at 110 and 275 mg/kg MEQ groups. The changed levels of ALT, AST, CREA, BUN, UA, Na, and K were noted in the serum of rats. The histopathologic examination showed that MEQ induced toxicity in the liver, kidney, adrenal, uterus and testis. The no-observed-adverse-effect level (NOAEL) for reproduction toxicity of MEQ was 25 mg/kg diet. The malformations and severe maternal toxicity of MEQ caused adverse effects on the conceptus and embryo, which result in fetal malformations and fetal deaths. In summary, the present study showed that MEQ induced maternal, embryo and reproductive toxicities as well as teratogenicity in rats.

## Introduction

**FIGURE 1:**
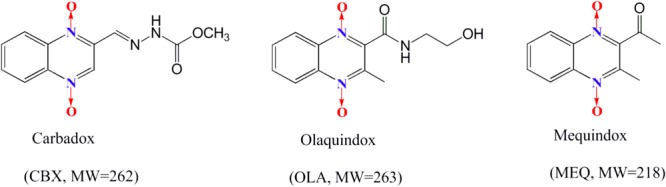
The chemical structures of carbadox, olaquindox, and mequindox.

As a class of synthetic heterocyclic agents, QdNOs are also known as DNA synthesis inhibitors and are usually used in human and veterinary medicine due to their broad range of biological properties including antibacterial, anti-candida, antitubercular, anticancer, antiprotozoal and growth promoting activities (Carta et al., 2005; Vicente et al., 2009; Wang et al., 2011a, 2016a; Cheng et al., 2015; Liu et al., 2017a). CBX and OLA were the classical members of QdNOs, which have been approved as an antibacterial drugs and growth-promoting agents for the improvement of weight gain and feed efficiency (WHO, 1991a,b) (**Figure 1**). MEQ (3-methyl-2-acetyl-*N*-1,4-dioxyquinoxaline, C_11_H_10_N_2_O_3_; MEQ) (**Figure 1**), a relatively new synthetic antibacterial agent developed for use in animal productions in China, considered as significant due to its antibacterial properties and growth promoting ability (Liu et al., 2012, 2017d; Wu et al., 2012; Li et al., 2014).

The use of MEQ as an animal feed additives or antimicrobial agents had caused serious health hazard effects. MEQ had adverse effects on pigs and chicken in clinical use (Chun, 2005; Hongxia et al., 2005; Ihsan, 2011). Chronic studies of MEQ on rats induced adrenal toxicity (Huang et al., 2009), invoked oxidative stress in liver and kidney (Huang et al., 2010b), and caused hepatic histological changes (Ihsan et al., 2010). Recent studies revealed that toxicity of liver, kidney, and testis in mice was induced by MEQ at a dose level of 25, 55, and 110 mg/kg diet (Liu et al., 2017b,c,d, 2018a). In genotoxicity tests, MEQ exhibited a strong genotoxic potential to bacteria, mammalian cells *in vitro* and *in vivo* and its mutagenicity is slightly higher than CBX (Ihsan et al., 2013a; Liu et al., 2016). MEQ caused chromosomal aberrations in V79 cells and micronucleus formation in mice (Wang et al., 2011a,b; Liu et al., 2016). Together with genotoxic findings of MEQ, two primary metabolites of MEQ were concluded to be genotoxic in a battery of four different short-term tests, including mouse lymphoma assay (MLA), Ames test, chromosomal aberration assay and bone marrow erythrocyte micronucleus assay (Liu et al., 2016, 2017d). A recent study suggested that MEQ induced genotoxicity and carcinogenicity in mice (Liu et al., 2018b). However, despite many years of usage in food producing animals, the toxic characteristics of MEQ, including its major toxic effects on the reproduction and development, had not been adequately investigated.

The literature regarding the reproductive toxicity and teratogenic potential of QdNOs was published previously (WHO, 1991a,b; Yoshimura, 2002; Ihsan et al., 2013a,b), which demonstrated that CBX and OLA were suspected to have developmental and reproductive toxicities with mutagenicity and carcinogenicity. The decrease in fetal and maternal body weights, and teratogenic effects were reported on days 8–15 of pregnancy in rats after exposure to CBX at doses as low as 25 mg/kg per day (Yoshimura, 2002). It was concluded that maternally toxic doses of CBX caused embryo-toxicity and embryo-lethality (JECFA, 2003a). OLA resulted in the histopathological alterations in testes of rats after administration at a dose of 5 mg/kg b.w./day (WHO, 1991b). Due to these undesirable effects, the use of CBX and OLA in food-producing animals was prohibited by the Health Canada and Commission of the European Community (Wu et al., 2007; Nasr et al., 2013). In 2001, Japan also prohibited use of OLA in animal feed (Zhao et al., 2008). MEQ was reported to induce endocrine and reproductive toxicity in Wistar rats at a dose range from 25 to 275 mg/kg diet (Ihsan et al., 2011). Recently, the testis toxicity was observed in mice after exposure to MEQ (25, 55, and 110 mg/kg) for 18 months (Liu et al., 2017b,c). These studies only focus on the testis toxicity, which can’t provide a comprehensive assessment of reproductive toxicity of MEQ. Though it has been widely applied in pigs and chickens in China since the 1980s (Liu et al., 2017d), no risk assessment on its reproductive and developmental toxicity was reported.

Most current international guidelines require a two generation reproduction test to evaluate the reproductive toxicity of industrial chemicals. Because of no reports of reproductive and development studies of MEQ, it is impossible to determine the NOAEL of developmental or reproductive and evaluate the risk of MEQ. For a long-term perspective, increased use of MEQ without comprehensive safety evaluation is questionable, especially the concerns related to the potential impact on human health of the proposed use in food producing animals. Therefore, the present study was designed to evaluate the two generation reproduction of MEQ in Wistar rats with a wide range of doses according to the following principles (FDA, 2000a,b; OECD, 2001 TG 416) and in line with GLP principles. This study would reveal the effects of long-term treatment of MEQ on animal reproductive organ and fertility. The achievements would not only provide a complete toxicity spectrum of MEQ, but also, offer scientific information for further risk evaluation of MEQ in food animals.

## Materials and Methods

### Test Substance

Mequindox (C_11_H_10_N_2_O_3_, purity 98%) was obtained from Beijing Zhongnongfa Pharmaceutical Co. Ltd. (Huanggang, China). All other reagents were of analytical grade.

### Animals and Husbandry

One hundred twenty five male and 125 nulliparous female Wistar rats (*Rattus norvegicus*, 5–6-wk old) were obtained from Center of Laboratory Animals of Hubei Province, Wuhan, China. The study was approved by the Ethical Committee of the Faculty of Veterinary Medicine (Huazhong Agricultural University, Wuhan, China). Males and females were acclimated to the laboratory for 7 days prior to the start of the experiment. Male and female rats found to be in good health were selected for use. The rats, five per cage per sex, were kept in an environment maintained at 20∼25°C, relative humidity of 40∼70% and a 12-h light: 12-h dark cycle. Beddings (Hardwood shavings) were used after sterilization by autoclaving. Feed and tap water were provided *ad libitum*. After quarantine, rats were randomly assigned to different groups by body weight (both sexes). Mated females were housed individually in shoebox cages containing nesting material throughout gestation and lactation. In the present study, the use of rats was in accordance with NIH Publication 85–23 “Guide for the Care and Use of Laboratory Animals” (NRC 2004). All animal studies were conducted in compliance with the guidelines for the Care and Use of Laboratory Animals of Hubei Provincial Laboratory Animal Public Service Center (permit number SYXK 2013-0044), and the protocol was approved by the Ethics Committee of Huazhong Agricultural University.

### Diet Preparation

Mequindox was incorporated in diets containing 0, 55, 110, or 275 mg/kg, equivalent to 0, 10, 20, or 50% of the LD50 in diets. A dose of 55 mg/kg diet was selected because it is the normal concentration of OLA permitted in food. Two hundred seventy five milligrams per kilogram dietary level MEQ inhibited feed intake and body weight of the rats (Ihsan et al., 2010), and therefore, it was used as the high dose in the present study. The medium dosage of 110 mg/kg and the minimal dosage of 25 mg/kg are within 2 and 0.5 times of the dose (55 mg/kg), respectively. Principle of dose selection was according to the U. S. Food and Drug Administration Toxicological Principles for the Safety Assessment of Food Ingredients IV.C.9.a. and IV.C.9.b. (FDA, 2000a,b). Therefore, the dose level of 25, 55, 110, and 275 mg/kg was selected in this study. MEQ was administered to rats by feeding in diet with the concentrations of 0, 25, 55, 110, and 275 mg/kg diet (Rat Maintenance Feed, Ground Fine from the Center of Laboratory Animals of Hubei Province, Wuhan, China) throughout the whole period (including exposure, mating, gestation, parturition and lactation periods). The diets were mixed separately by group and placed in adequately labeled dark containers. The diets were prepared weekly and analyzed periodically for verification of concentration, homogeneity and stability. This study was conducted in compliance with FDA Good Laboratory Practice Regulations (Part 58 of 21 CFR) (FDA, 2010).

### Experimental Design

The two generation feeding reproduction study and teratogenic study were performed according to FDA Toxicological principles for the safety assessment of food ingredients. IV.C.9.a. Guidelines for Reproduction Toxicity Studies (FDA, 2000a) and IV.C.9.b. Guidelines for Developmental Toxicity Studies (FDA, 2000b), respectively.

**FIGURE 2:**
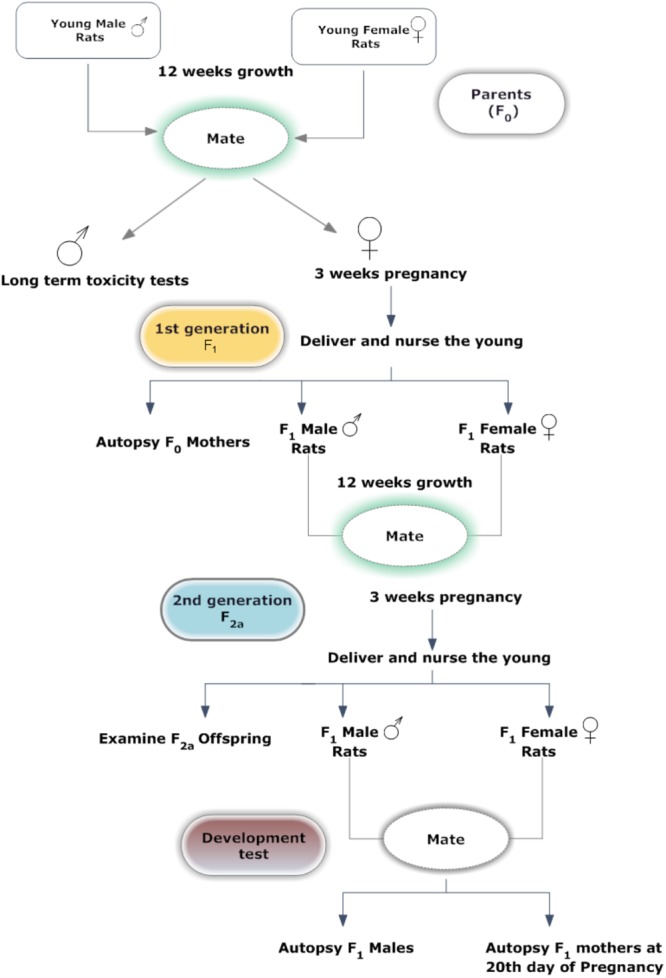
Experimental design for two generation reproductive and teratogenic test.

A graphic representation of the study design is presented in **Figure 2**. After acclimation, 25 F0 rats were randomly assigned to five groups. Each group of rats were fed with one of the following five diets; control diet, four basal diets mixed with MEQ (25, 55, 110, and 275 mg/kg) for 12 weeks prior to the mating period. Twenty-five male and 25 female F1 weanlings were selected as F1 parents with at least one male and one female in each litter on PND 21–25. F1 selected rats were administered MEQ in the diet of the particular formulations in the same manner as described for F0 rats. Administration of MEQ in the diet was continued throughout the mating, gestation and lactation periods. Unselected F1 weanlings and all F2a weanlings were killed on PND 25. After weaning of F2a generation, 25 F1 females and 25 males from each group were selected and given rest for 10 days. After 10 days, these rats were bred again to get F2b generation with same conditions as mentioned above. On GD 20, pregnant female rats were subjected to cesarean section for teratogenic examination.

### Mating Procedures

For mating, one female and one male were placed into one cage overnight. Successful mating was ascertained by the presence of sperm in the vaginal smear, and the following first 24 h was designated as day 0 of gestation. Mated females were housed singly in clear polycarbonate cages with stainless steel wire lids and were allowed tap water and feed *ad libitum*.

After 12 weeks pre-breed period, F0 and F1 rats were housed as breeding pairs with the ratio of 1:1, until evidence of mating was observed or for 21 consecutive days. Rats in the F1 generation were bred twice to produce F2a and F2b generations. Copulation was examined every morning and successful mating was confirmed by the presence of vaginal plug. Mated females were housed singly in clear polycarbonate cages with stainless steel wire lids and were allowed tap water and feed *ad-libitum*. F0 and F1 females that did not mate were cohabited with another male from the same group who had been proven to copulate. For F1 mating, cohabitation of siblings was avoided.

### Parameters Evaluated

#### Parental Data in the Teratogenic Study

Routine cage-side observations were performed on all animals twice a day throughout the study for general signs of toxicologic and pharmacologic effects, morbidity and mortality, general appearance, and behavior. An expanded set of clinical evaluations, performed outside of the cage, was performed on all animals twice before test material administration and weekly during the study period to detect neurologic disorders, behavioral changes, autonomic dysfunctions, and other signs of nervous system toxicity. Feed intakes of rats were measured weekly. Maternal body weights were measured on GD 0, 7, 14, and 21. Maternal body weight gains were calculated. Throughout gestation, all pregnant rats were observed daily for mortality, morbidity, general appearance and behavior. Females were sacrificed and subjected to external and internal macroscopic examination at the scheduled termination (GD 21). The day of parturition was designated as PND 0. Total litter size and number of live and dead pups were recorded. The live pups were counted, sexed, weighed, and examined grossly on PND 0, 4, 7, 14, and 21.

#### Parental Data in the Reproduction Study

Each rat was observed at least twice daily throughout the study. Relevant behavioral changes and all signs of toxicity, morbidity, or mortality were recorded. Estrous cycle length and normality were observed daily by vaginal smears for all F0 and F1 females during a minimum of 3 weeks before mating and during cohabitation. The duration of gestation was calculated from day 0 of pregnancy. The mating index [(number of males (or females) with evidence of mating/total number of males (or females) paired) × 100], fertility index [number of females with a confirmed pregnancy/total number of females paired) × 100], live birth rate [(number of females producing live litter/total number of pregnancy) × 100], survival rate of birth [(Survival number of litter on the forth days of birth/total number of surviving litter at birth) × 100], survival rate of lactation (%) [(Survival number of weaned litter on the 21st days of birth/total number of surviving litter at birth) × 100], and sex ratio (expressed as % males per litter) were calculated. Throughout gestation, all pregnant rats were observed daily for mortality, morbidity, general appearance, and behavior. Females were sacrificed and subjected to external and internal macroscopic examination at the scheduled termination (GD 21 or pups weaned).

For the F_0_ and F_1_ generation males selected for mating, sperm samples obtained from the right cauda epididymis per testis were collected and then placed in Dulbecco’s phosphate buffered saline (maintained at approximately 37°C) with 10 mg/mL bovine serum ALB. After 10-min incubation, sperm motility was determined under light microscopy (Olympus BX 41, Japan). Sperm (minimum 200 per sample) samples from the cauda epididymis were examined as a fixed wet preparation and classified as either normal (both head and midpiece appear normal) or abnormal (i.e., fusion, isolated heads, misshapen heads, and/or tails).

#### Cesarean Section in the Teratogenic Study

The dams were euthanized on GD 21. The ovaries and uterus of each rat were removed and the status of all implantation sites, i.e., live and dead fetuses, early and late resorptions, and total implantations were examined. Live fetuses were weighed individually. All live fetuses were sexed, weighed, and inspected for external malformations; including cleft palate. Approximately one-half of the live fetuses in each litter were randomly selected for either skeletal or visceral examination. The skeletal evaluation of 85% ethanol-fixed fetuses was performed after staining the skeleton with Alizarin Red S and clearing with the potassium hydroxide solution. The remaining live fetuses in each litter were fixed in Bouin’s solution prior to dissection. A freehand razor sectioning technique was adapted to detect internal malformations of the head and abdomen.

#### Growth of Offspring in the Reproduction Study

In each litter, the number of pups, stillbirths, live births, and the presence of gross anomalies were examined after delivery. Dead pups were necropsied and observed for gross defects. The neonates were carefully observed, and their sex and weight were noted on PND 0, 4, 7, 14, and 21.

#### Clinical Chemistry

Serum chemistry was examined using Synchron Clinical System CX4 (Beckman Coulter, Brea, CA, United States) according to the manufacturer’s directions (Beijing Leadman Biochemistry Technology Co. Ltd, Beijing, China). Parameters in serum chemistry included ALB, ALT, AST, TP, CREA, CL^-^, ALP, UA and so on.

#### Macroscopic Examination, Organ Weight, and Relative Organ Weight

The macroscopical examination of organs/tissues included the visible lesions on the external surface. Each rat received a gross necropsy and microscopic examination of standard tissues including: liver, spleen, kidney, adrenal, testes, epididymis, uterus, and ovary. The organs/tissues were carefully examined and gross lesions were recorded. During necropsies, a complete list of organs and tissues was weighed separately to calculate the organ weights per 100 g body weight (relative organ weight).

#### Pathology Examinations

All the reproductive organs, including testis, epididymis, prostate, uterus and ovaries were examined macroscopically, and gross lesions were recorded. The tissues from each rat, with the exception of testis, were preserved in 10% neutral-buffered formalin and slides prepared for histopathological examination. Routine paraffin embedding technique was used for histological examination of tissue sections. Sections of 5 μm thickness stained with H&E were examined under light microscopy (Olympus BX 41, Japan) for morphological changes. As a necessary step to determine a no-observed-adverse-effect-level (NOAEL) in target organs, other tissues such as liver and kidneys from treatment groups were also examined histologically.

### Statistical Analyses

For pre-breed data, Levene’s test was performed to determine whether the groups had equal variances. If the variances were homogeneous, ANOVA was carried out. If not, they were analyzed by the Kruskal–Wallis non-parametric ANOVA. If either of the tests showed a significant difference among the groups, the data were analyzed by the multiple comparison procedure of the Dunnett’s *post hoc* test or Mann–Whitney *U* test.

Continuous data such as maternal body weight, food consumption, fetal body weight, sperm numbers, body length and tail length were subjected to ANOVA, and Dunnett’s multiple comparison tests were conducted when analytic results were significant. Incidence data such as the gender ratio, external, skeletal and visceral variations, the proportions of litters with malformations and developmental variations were compared using a chi-square test and Fisher’s exact test. Sperm motility and sperm morphology were analyzed with Kruskal-Wallis test with Mann-Whitney *U* test. The unit of comparison was the pregnant dam or the litter for data of the gestational period and thereafter. A difference was considered statistically significant at *p*< 0.01 or *p*< 0.05.

## Results

### Clinical Observations

All the F0 male and female rats were survived except three males and one pregnant female were found dead during pre-breeding and mating periods at 275 mg/kg MEQ diet. The rats were weak, emaciated, and showed some neurological signs such as hyperactive, readily irritated and aggressive on handling at the 275 mg/kg MEQ mg/kg group.

### Body Weight During the Pre-mating, Mating, Gestation, and Lactation Periods

**FIGURE 3:**
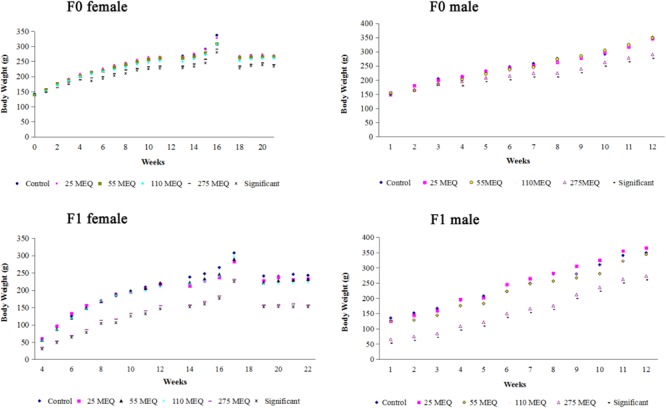
Mean body weights of F0 female, F0 male, F1 female, and F1 male in the two generation reproductive and teratogenic test.

The body weights of F_0_ and F_1_ male and female rats from pre-bred, mating, gestation and lactation periods are presented in **Figure 3**. The body weights of F0 females were significantly reduced at 275 mg/kg MEQ from weeks 5 to 12. Compared with control group, body weights of F1 females from weeks 1 to 12 after weaning were significant decreased at 275 mg/kg MEQ group (**Figure 3**). Body weights of F2 on day 21 after birth decreased significantly at 275 mg/kg MEQ when compared with controls.

For males, there is a downward trend in body weight of F0 males at the 25, 55, and 110 mg/kg MEQ groups during most of weeks (except at first and ninth weeks). For F0 males, a significantly reduced body weight was noted at 275 mg/kg MEQ group from weeks 4 to 12. A reduced body weight of F_1_ males was observed at 55 mg/kg MEQ group from weeks 1 to 3. Body weights of F_1_ males were significantly decreased from 1 to 12 weeks at 275 mg/kg MEQ group when compared with controls. Decreased body weights from 1 to 5 weeks and 10 to 12 weeks were noted in F_1_ males at 55 and 110 mg/kg MEQ groups.

### Reproductive Effects (F0 Parents/F1 Offspring and F1 Parents/F2 Offspring)

**Table 1** presented the reproductive parameters of the F0 parent/F1 offspring. MEQ reduced the number of pregnant females, live pups, litter loss and sex ratio in the live pups at 275 mg/kg MEQ diet as compared to the control. Three dams experienced total litter loss by day 1∼4 of the lactation at 275 mg/kg MEQ group. The mating and fertility indices were significantly decreased at 110 and 275 mg/kg groups. Compared to control group, there is a downward trend in body weight of F1 pups at the 55, 110, and 275 mg/kg groups. The survival rate of birth was significantly reduced in F1 pups at the 275 mg/kg group.

**Table 1:** Summery of F0 generation mating and F1 litter and survival data (Mean ± SD).

	Control	M25	M55	M110	M275
No of rats (male/female)	25/25	25/25	25/25	25/25	22/25
No. of pregnant females	24	23	23	20	19^∗^
Mating index (%)	96	88	92	80^∗∗^	76^∗∗^
Fertility index (%)	96	88	92	80^∗∗^	76^∗∗^
Gestation index	100	95.65	91.30	100	94.74
Total no. of pups/litter	9.38 ± 2.19	9.4 ± 3.25	9.46 ± 2.21	8.57 ± 3.88	9.57 ± 2.17
No. of live pups/litter	9.38 ± 2.19	9.27 ± 3.17	9.46 ± 2.21	8.57 ± 3.88	8.21 ± 3.14^∗^
Sex ratio (%) of F1 pups	50.7	48.2	48.1	46.6	42.5^∗^
**Pup viability index during lactation (%)**		
Live birth rate (%)	100	98.6	100	93.3	88.1
Survival rate of birth (%)	92.9	95.4	82.2	83.8	56.6^∗∗^
Survival rate of lactation (%)	98.0	99.1	94.6	89.4	93.4
No. of total litter losses	0	0	1	2	4^∗^
Mean pup body weights day 1 (g)	6.30 ± 0.95	6.33 ± 0.57	5.91 ± 0.71	6.26 ± 0.66	5.40 ± 0.69
Mean pup body weights day 4 (g)	12.47 ± 1.65	11.9 ± 1.67	10.09 ± 1.13	10.06 ± 2.02	8.11 ± 1.63
Mean pup body weights day 7 (g)	17.0 ± 1.7	16.9 ± 1.5	15.9 ± 2.1	15.3 ± 1.6	14.12 ± 2.5
Mean pup body weights day 14 (g)	23.51 ± 3.09	24.6 ± 4.32	23.19 ± 3.64	20.95 ± 2.92	19.40 ± 2.54
Mean pup body weights day 21 (g)	36.87 ± 5.30	39.2 ± 7.55	34.35 ± 4.43	32.29 ± 4.84	22.42 ± 4.28

*SD, standard deviation; M, mequindox; M25, 25 mg/kg diet; M55, 55 mg/kg diet; M110, 110 mg/kg diet; M275, 275 mg/kg diet.*

*^∗^Significantly different from control values at *P* ≤ 0.05.*

*^∗∗^Significantly different from control values at *P* ≤ 0.01.*

The reproductive parameters of F1 parent/F2 offspring were shown in **Table 2**. The reduced number of pregnant females, live pups, litter loss and total number of pups was noted at 275 mg/kg F1 females, as compared to the control. The increased sex ratio of F2 pups was noted at 25 mg/kg group. There is a downward trend in body weight of F2 pups at the 110 and 275 mg/kg groups. The mating and fertility indices of F1 were significantly decreased at 110 and 275 mg/kg groups. One dam experienced total litter loss by day 4∼21 of lactation in the 25 mg/kg MEQ group and by day 5 of lactation at 55 mg/kg MEQ group. At 110 and 275 mg/kg MEQ groups, three dams experienced the total litter loss by day 5∼21 of lactation. No clear clinical signs of toxicity were observed in these dams with total litter loss.

**Table 2:** Summery of F1 generation mating and F2 litter and survival (Mean ± SD).

	Control	M25	M55	M110	M275
No of rats (male/female)	25/25	25/25	25/25	25/25	25/25
No. of pregnant females	23	20	21	18	14^∗^
Mating index (%)	91.7	83.3	87.5	75^∗∗^	58.3^∗∗^
Fertility index (%)	91.7	83.3	87.5	75^∗∗^	58.3^∗∗^
Gestation index	95.46	100	95.24	100	100
Total no. of pups/litter	10.17 ± 2.44	8.94 ± 2.18	9.0 ± 2.69	9.12 ± 3.04	7.46 ± 1.90^∗^
No. of live pups/litter	10.17 ± 2.44	8.67 ± 2.91	8.53 ± 2.92	8.35 ± 2.89	7.38 ± 1.89^∗^
Sex ratio (%) of F2 pups	50.82	58.39^∗^	47.92	51.41	45.36
**Pup viability index during lactation (%)**		
Live birth rate (%)	99.8	90.5	87.87^∗^	93.1	78.8^∗∗^
Survival rate of birth (%)	97	97.9	79.3^∗∗^	85.2^∗^	78.0^∗∗^
Survival rate of lactation (%)	100	82.9	69.6^∗^	78.2^∗∗^	93.8
No. of total litter losses	0	1	2	3	3^∗^
Mean pup body weights day 1 (g)	5.99 ± 0.83	6.17 ± 0.88	6.19 ± 0.68	5.93 ± 0.82	5.62 ± 1.03
Mean pup body weights day 4 (g)	13.15 ± 2.30	11.14 ± 0.94	12.4 ± 2.07	11.89 ± 1.44	13.03 ± 0.98
Mean pup body weights day 7 (g)	17.1 ± 1.91	17.4 ± 1.82	17.0 ± 1.7	15.5 ± 1.22	15.1 ± 1.71
Mean pup body weights day 14 (g)	22.1 ± 2.98	19.0 ± 3.21	20.9 ± 3.4	21.72 ± 3.22	20.02 ± 1.73
Mean pup body weights day 21 (g)	31.38 ± 6.05	30.52 ± 7.2	32.02 ± 5.44	28.2 ± 7.03	30.47 ± 2.56

*SD, standard deviation; M, mequindox; M25, 25 mg/kg diet; M55, 55 mg/kg diet; M110, 110 mg/kg diet; M275, 275 mg/kg diet.*

*^∗^Significantly different from control values at *P* ≤ 0.05.*

*^∗∗^Significantly different from control values at *P* ≤ 0.01.*

### Biochemical Changes (F0 Parents and F1 Parents)

The results of serum clinical chemistry of F0 and F1 parents were presented in **Table 3**. For F0 parents, a significant changed levels of AST, CREA, UA, Na, and K were noted in 275 mg/kg MEQ group. Significant changed biochemical index were found for ALT, AST, TP, Na, and K at 275 mg/kg F1 parent group. The trend of rising levels of ALT, TP, CREA, and K, and a downward trend in BUN and K were found in F0 and F1 parent at all the tested groups.

**Table 3:** Serum clinical chemistry parameters of F0 and F1 Wistar rats.

Parameters	Control	M25	M55	M110	M275
**F0**		
ALT (IU/L)	59.11 ± 11.03^a^	76.31 ± 30.52	67.76 ± 11.10	78.5 ± 35.25	79.41 ± 18.51
AST (IU/L)	121.0 ± 21.83	121.6 ± 25.51	150.0 ± 30.80	141.6 ± 27.31	243.7 ± 61.67^∗^
ALP (IU/L)	35.71 ± 7.03	46.10 ± 14.85	35.51 ± 7.92	48.1 ± 15.58	49.82 ± 8.61
TP (g/L)	71.20 ± 2.96	73.32 ± 5.28	73.22 ± 5.26	75.31 ± 5.62	82.13 ± 8.48
ALB (g/L)	34.50 ± 3.14	31.31 ± 1.64	35.41 ± 3.29	33.32 ± 1.45	33.81 ± 1.74
CREA (mmol/L)	48.90 ± 7.32	53.60 ± 7.65	50.01 ± 9.72	55.51 ± 9.56	59.75 ± 9.34^∗^
BUN (mmol/L)	8.9 ± 0.88	7.6 ± 1.29	7.7 ± 0.41	7.8 ± 1.21	7.7 ± 0.23
UA (μmol/L)	132.5 ± 27.63	113.8 ± 9.61	126.3 ± 14.44	115.8 ± 11.56	105.6 ± 16.6^∗^
Na (mmol/L)	126.2 ± 2.84	124.0 ± 2.38	131.7 ± 3.68^∗∗^	126.0 ± 2.83	120.7 ± 2.63^∗∗^
K (mmol/L)	4.3 ± 0.63	4.9 ± 1.09	5.4 ± 0.94	5.9 ± 0.89	6.4 ± 0.86^∗∗^
CL (mmol/L)	104.0 ± 3.08	101.9 ± 2.25	105.6 ± 4.5	104.9 ± 2.52	105.7 ± 8.9
**F1**		
ALT (IU/L)	78.50 ± 9.07	87.42 ± 12.34	80.31 ± 14.56	89.5 ± 12.97	132.9 ± 28.61^∗^
AST (IU/L)	173.0 ± 30.07	177.5 ± 46.1	171.7 ± 48.71	179.7 ± 46.6	231.3 ± 43.74^∗^
ALP (IU/L)	81.71 ± 11.08	73.01 ± 14.23	73.92 ± 10.91	76.0 ± 14.01	73.41 ± 16.24
TP (g/L)	75.61 ± 2.75	79.13 ± 2.15	80.9 ± 2.08	81.22 ± 4.06	91.24 ± 9.41^∗^
ALB (g/L)	33.30 ± 3.83	34.53 ± 2.34	34.4 ± 1.61	36.72 ± 1.59	37.72 ± 1.19
CREA (mmol/L)	50.30 ± 4.7	57.42 ± 5.32	54.4 ± 6.43	59.61 ± 5.71	67.61 ± 8.13
BUN (mmol/L)	8.6 ± 1.07	7.3 ± 0.12	8.2 ± 0.55	7.5 ± 0.98	7.6 ± 1.23
UA (μmol/L)	94.70 ± 15.71	90.42 ± 16.54	91.81 ± 31.18	91.91 ± 16.23	90.31 ± 20.04
Na (mmol/L)	158.5 ± 3.64	151.3 ± 4.38	151.5 ± 2.65	153.5 ± 4.88	141.8 ± 2.62^∗∗^
K (mmol/L)	6.6 ± 0.94	7.1 ± 3.25	6.9 ± 1.61	7.5 ± 3.14	9.7 ± 2.38^∗∗^
CL (mmol/L)	101.8 ± 8.87	105.6 ± 1.82	102.8 ± 4.44	107.7 ± 1.19	104.9 ± 2.94

*SD, standard deviation. M, mequindox; M25, 25 mg/kg diet; M55, 55 mg/kg diet; M110, 110 mg/kg diet; M275, 275 mg/kg diet.*

*^*a*^Data presented as mean ± standard deviation of 10 animals per sex group.*

*^∗^Significantly different from control group at *P* < 0.05.*

*^∗∗^Significantly different from control group at *P* < 0.01.*

### Organ Weights and Relative Organ Weights (F0 and F1 Animals)

At sacrifice, the body weights and organ weights of F0 and F1 females were weighed. At 275 mg/kg MEQ group, significant changes of organ weights of liver, spleen, adrenal, ovary, and uterus were found in F0 females (**Table 4**); significant decrease of organ weights of liver, kidneys, and ovary were noted in F1 females (**Table 5**). For F0 females, significant increase in the relative organ weights of brain, liver, spleen, and uterus were noted at 275 mg/kg MEQ group, while significant decrease in the relative organ weights of adrenals and ovary were found at 275 mg/kg MEQ group (**Table 4**). For F1 females, significant increase in the relative organ weights of brain, liver, and spleen were observed at 275 mg/kg MEQ group, while significant decrease in the relative organ weights of ovary were noted at 275 mg/kg MEQ group (**Table 5**).

**Table 4:** Organ weight (g) of F0 female Wistar rats.

Organs	Control	M25	M55	M110	M275
Final body	268.0 ± 20.6	270.4 ± 47.58	266.6 ± 38.52	261.0 ± 37.46	240.7 ± 33.53^∗^
Absolute (g)					
Brain	1.34 ± 0.09	1.34 ± 0.08	1.34 ± 0.08	1.31 ± 0.08	1.29 ± 0.09
Liver	5.83 ± 0.45^a^	7.11 ± 1.43^∗^	6.54 ± 1.42	6.75 ± 1.73	7.85 ± 1.47^∗^
Spleen	0.48 ± 0.10	0.51 ± 0.1	0.51 ± 0.12	0.52 ± 0.1	0.68 ± 0.12^∗^
Kidneys	0.76 ± 0.06	0.84 ± 0.16	0.73 ± 0.11	0.69 ± 0.15	0.81 ± 0.13
Adrenal	0.046 ± 0.011	0.041 ± 0.007	0.038 ± 0.007	0.033 ± 0.009^∗^	0.025 ± 0.010^∗^
Uterus	3.12 ± 1.97	3.74 ± 1.68	3.90 ± 1.83	3.73 ± 1.89	2.35 ± 1.73^∗^
Ovary	0.38 ± 0.16	0.29 ± 0.17	0.31 ± 0.26	0.26 ± 0.13	0.11 ± 0.32^∗^
**Relative (g/100g b.w.)**					
Brain	0.44 ± 0.04	0.45 ± 0.04	0.48 ± 0.04^∗^	0.55 ± 0.04^∗∗^	0.56 ± 0.05^∗∗^
Liver	2.50 ± 0.18	2.67 ± 0.35	2.72 ± 0.41	3.12 ± 0.68^∗^	3.23 ± 0.34^∗^
Spleen	0.21 ± 0.03	0.20 ± 0.03	0.21 ± 0.03	0.21 ± 0.03^∗^	0.28 ± 0.04^∗^
Kidneys	0.33 ± 0.04	0.31 ± 0.04	0.31 ± 0.03	0.32 ± 0.05	0.33 ± 0.03
Adrenal	0.019 ± 0.007	0.016 ± 0.005	0.016 ± 0.004	0.015 ± 0.003	0.009 ± 0.004^∗^
Uterus	1.26 ± 0.23	1.19 ± 0.32	1.06 ± 0.34	1.23 ± 0.32	1.72 ± 0.25^∗∗^
Ovary	0.07 ± 0.09	0.07 ± 0.02	0.06 ± 0.01	0.08 ± 0.04	0.05 ± 0.05^∗^

*SD, standard deviation; M, mequindox; M25, 25 mg/kg diet; M55, 55 mg/kg diet; M110, 110 mg/kg diet; M275, 275 mg/kg diet.*

*^*a*^Data presented as mean ± standard deviation of 20 animals per group.*

*^∗^Significantly different from control group at *P* < 0.05.*

*^∗∗^Significantly different from control group at *P* < 0.01.*

**Table 5:** Organ weight (g) of F1 female Wistar rats.

Organs	Control	M25	M55	M110	M275
Final body	301.8 ± 33.2	298.6 ± 46.3	264.6 ± 36.9^∗∗^	268.0 ± 42.5^∗∗^	205.1 ± 27.9^∗∗^
Absolute (g)					
Brain	1.49 ± 0.07	1.52 ± 0.05	1.50 ± 0.07	1.46 ± 0.11	1.48 ± 0.07
Liver	8.87 ± 1.95^a^	8.40 ± 2.18	7.17 ± 1.88^∗^	8.31 ± 2.33	7.10 ± 1.90^∗∗^
Spleen	0.64 ± 0.22	0.56 ± 0.12	0.54 ± 0.13	0.55 ± 0.17	0.54 ± 0.11
Kidneys	0.81 ± 0.24	0.82 ± 0.21	0.78 ± 0.23^∗^	0.76 ± 0.23^∗^	0.65 ± 0.16^∗∗^
Adrenal	0.047 ± 0.033	0.040 ± 0.015	0.050 ± 0.060	0.065 ± 0.122	0.028 ± 0.008
Ovary	0.22 ± 0.11	0.21 ± 0.09	0.15 ± 0.05^∗^	0.19 ± 0.10	0.13 ± 0.04^∗∗^
**Relative (g/100g b.w.)**					
Brain	0.29 ± 0.02	0.30 ± 0.02	0.30 ± 0.03	0.34 ± 0.03^∗∗^	0.36 ± 0.03^∗∗^
Liver	2.92 ± 0.40	2.79 ± 0.39	2.72 ± 0.39	3.07 ± 0.51^∗^	3.45 ± 0.72^∗∗^
Spleen	0.21 ± 0.07	0.19 ± 0.03	0.21 ± 0.05	0.21 ± 0.05	0.27 ± 0.05^∗∗^
Kidneys	0.30 ± 0.07	0.28 ± 0.09^∗^	0.29 ± 0.08	0.28 ± 0.07	0.32 ± 0.09
Adrenal	0.015 ± 0.010	0.013 ± 0.004	0.021 ± 0.030	0.023 ± 0.037	0.014 ± 0.005
Ovary	0.037 ± 0.016	0.036 ± 0.017	0.028 ± 0.008	0.034 ± 0.015	0.032 ± 0.010^∗^

*SD, standard deviation; M, mequindox; M25, 25 mg/kg diet; M55, 55 mg/kg diet; M110, 110 mg/kg diet; M275, 275 mg/kg diet. ^*a*^Data presented as mean ± standard deviation of 20 animals per group. ^∗^Significantly different from control group at *P* < 0.05. ^∗∗^Significantly different from control group at *P* < 0.01.*

For males, significant decrease in organ weights of brain, kidneys, adrenal, testis, and epididymis were observed in 275 mg/kg MEQ F0 male group (**Table 6**). Significant reduced of organ weights of brain, kidneys, and epididymis were noted at 275 mg/kg MEQ F1 males (**Table 7**). Significant increase in the relative organ weights of liver and testis were found at 275 mg/kg group in F0 and F1 males (**Tables 6, 7**).

**Table 6:** Organ weight (g) of F0 male Wistar rats.

Organs	Control	M25	M55	M110	M275
Final body	390.53 ± 36.17	391.37 ± 33.32	404.57 ± 42.85	402.12 ± 65.46	335.64 ± 40.57^∗∗^
Absolute (g)					
Brain	1.77 ± 0.19^a^	1.88 ± 0.16	1.78 ± 0.25	1.48 ± 0.14^∗∗^	1.43 ± 0.12^∗∗^
Liver	10.1 ± 1.27	9.94 ± 1.43	10.98 ± 1.89	11.15 ± 2.37	9.63 ± 1.26
Spleen	0.77 ± 0.17	0.76 ± 0.16	0.85 ± 0.37	0.75 ± 0.10	0.71 ± 0.16
Kidneys	1.23 ± 0.19	1.22 ± 0.09	1.24 ± 0.13	1.20 ± 0.23	1.03 ± 0.14^∗^
Adrenal	0.048 ± 0.020	0.043 ± 0.016	0.034 ± 0.006^∗^	0.032 ± 0.009^∗∗^	0.030 ± 0.005^∗∗^
Testis	3.46 ± 0.33	3.36 ± 0.30	3.20 ± 0.42	3.30 ± 0.27	2.79 ± 0.37^∗^
Epidedmius	1.37 ± 0.31	1.35 ± 0.21	1.36 ± 0.23	1.30 ± 0.22	1.12 ± 0.32^∗^
**Relative (g/100g b.w.)**					
Brain	0.35 ± 0.03	0.37 ± 0.03	0.36 ± 0.03	0.34 ± 0.02	0.35 ± 0.03
Liver	2.58 ± 0.25	2.53 ± 0.23	2.71 ± 0.38	2.77 ± 0.32	2.87 ± 0.38^∗^
Spleen	0.20 ± 0.05	0.19 ± 0.04	0.21 ± 0.09	0.19 ± 0.03	0.21 ± 0.04
Kidneys	0.32 ± 0.04	0.31 ± 0.02	0.31 ± 0.04	0.30 ± 0.02	0.31 ± 0.03
Adrenal	0.012 ± 0.005	0.011 ± 0.004	0.009 ± 0.001	0.008 ± 0.002	0.009 ± 0.002
Testis	0.87 ± 0.021	0.87 ± 0.017	0.88 ± 0.036	0.87 ± 0.025	1.02 ± 0.036^∗∗^
Epidedmius	0.35 ± 0.06	0.30 ± 0.06	0.34 ± 0.05	0.33 ± 0.06	0.33 ± 0.08

*SD, standard deviation; M, mequindox; M25, 25 mg/kg diet; M55, 55 mg/kg diet; M110, 110 mg/kg diet; M275, 275 mg/kg diet.*

*^*a*^Data presented as mean ± standard deviation of 20 animals per group.*

*^∗^Significantly different from control group at *P* < 0.05.*

*^∗∗^Significantly different from control group at *P* < 0.01.*

**Table 7:** Organ weight (g) of F1 male Wistar rats.

Organs	Control	M25	M55	M110	M275
Final body	393.5 ± 30.4	390.5 ± 29.0	362.1 ± 32.8^∗^	401.4 ± 47.2	279.7 ± 41.8^∗^
Absolute (g)					
Brain	1.92 ± 0.16^a^	1.92 ± 0.12	1.90 ± 0.15	1.64 ± 0.10^∗∗^	1.57 ± 0.13^∗∗^
Liver	12.4 ± 1.16	12.3 ± 1.46	12.07 ± 1.47^∗^	13.06 ± 1.92	10.17 ± 1.99^∗^
Spleen	0.69 ± 0.10	0.66 ± 0.11	0.62 ± 0.10	0.66 ± 0.08	0.52 ± 0.10
Kidneys	2.31 ± 0.26	2.38 ± 0.31	2.26 ± 0.30	2.20 ± 0.24	1.67 ± 0.29^∗^
Adrenal	0.056 ± 0.022	0.056 ± 0.010	0.051 ± 0.010	0.068 ± 0.023^∗^	0.046 ± 0.008
Testis	3.38 ± 0.36	3.41 ± 0.41	3.65 ± 0.39	3.64 ± 0.79	3.57 ± 1.07
Epidedmius	1.10 ± 0.21	1.37 ± 0.64	1.04 ± 0.32	1.05 ± 0.13	0.79 ± 0.13^∗^
**Relative (g/100g b.w.)**					
Brain	0.38 ± 0.02	0.37 ± 0.02	0.38 ± 0.01	0.38 ± 0.02	0.38 ± 0.02
Liver	3.21 ± 0.29	3.19 ± 0.18	3.36 ± 0.29	3.27 ± 0.22	3.64 ± 0.38^∗^
Spleen	0.18 ± 0.02	0.17 ± 0.03	0.17 ± 0.02	0.17 ± 0.02	0.19 ± 0.03
Kidneys	0.60 ± 0.04	0.62 ± 0.08	0.63 ± 0.07	0.55 ± 0.05	0.60 ± 0.03
Adrenal	0.015 ± 0.006	0.015 ± 0.003	0.014 ± 0.002	0.017 ± 0.006	0.017 ± 0.004
Testis	0.87 ± 0.09	0.87 ± 0.11	0.91 ± 0.056	0.91 ± 0.13	1.05 ± 0.25^∗^
Epidedmius	0.29 ± 0.07	0.36 ± 0.18	0.29 ± 0.09	0.28 ± 0.03	0.27 ± 0.05

*SD, standard deviation. M, mequindox; M25, 25 mg/kg diet; M55, 55 mg/kg diet; M110, 110 mg/kg diet; M275, 275 mg/kg diet.*

*^*a*^Data presented as mean ± standard deviation of 20 animals per group.*

*^∗^Significantly different from control group at *P*< 0.05.*

*^∗∗^Significantly different from control group at *P* < 0.01.*

### Pathology Findings (F0 and F1 Animals)

**FIGURE 4:**
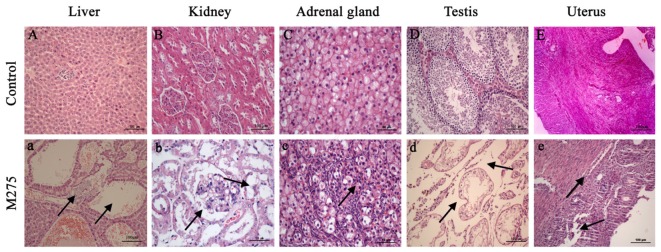
Selected microphotographs of liver, kidney, adrenal gland, testis and uterus (200X and 400X). M275, 275 mg/kg diet. **(A)** Liver (200X), **(B)** kidney (200X), **(C)** adrenal (400X), **(D)** testis (200X), and **(E)** uterus (200X) of F_0_ and F_1_ from the control group; **(a)** Liver in the 275 mg/kg MEQ group (200X). The vacuoles with a large number of blood cells, and hyperplasia of the epithelioid cells of the bile duct were marked with arrows; **(b)** Kidney in the 275 mg/kg MEQ group (400X). The swelling and hyperplasia of renal vesicle wall cell, and degeneration and necrosis of renal tubular epithelium were marked with arrows; **(c)** Adrenal gland in the 275 mg/kg MEQ group (400X). The proliferation of fascicular zone cell, increased binuclear cell and adrenocortical tumor were marked with arrows; **(d)** Testis in the 275 mg/kg MEQ group (200X). The broadening of interstitial, necrosis and dissolution of spermatogonial cells and spermatocytes in the lumen were marked with arrows; **(e)** Uterus in the 275 mg/kg MEQ group (200X). The incomplete structure and neutrophil infiltration in submucosal glands were marked with arrows.

As shown in **Figure 4**, obvious histopathological alterations were noted in F0 parents and F1 pups on day 28 at the 110 and 275 mg/kg MEQ groups: the degeneration and necrosis of the hepatocytes, and the hyperplasia of the epithelioid cells of the bile duct (**Figure 4A**); cells swelling, swollen, proliferated, degeneration and necrosis in many areas of kidney (**Figure 4B**); proliferation of fascicular zone cell, increased binuclear cell and adrenocortical tumor (**Figure 4C**). The pathology findings indicated the changed histopathological observations in testis and uterus at 110 and 275 mg/kg MEQ groups. Broadening of interstitial, necrosis and dissolution of spermatogonial cells and spermatocytes in the lumen were noted in testis (**Figure 4D**). The structure of the uterus is incomplete, and the neutrophil infiltration in submucosal glands (**Figure 4E**).

## Discussion

In present study, first time two-generation reproductive toxicity study was performed to further evaluate the potential effects of MEQ on reproduction of rats, which provided the information about adverse effects of MEQ on reproduction of rats.

In the two generation reproduction test, a significant decrease in the body weight from fifth week and a significant decrease in feed consumption in most days of week were observed at 275 mg/kg MEQ F0 female group. These results suggested that MEQ caused toxicity to the body weight and food consumption of rats. This finding was similar to a sub-chronic feeding test of MEQ, which demonstrated that the decreased feed consumption was accounted for the decrease in body weight (Ihsan et al., 2010).

In current study, a significant loss of body weight in females were found at 110 and 275 mg/kg MEQ groups, which indicated the maternal toxicity caused by MEQ. MEQ produced significant lower sex ratio in the live pups, viability indices and number of litter loss at 275 mg/kg MEQ diet as compared to the control. The indices of mating and fertility were significantly decreased in 110 and 275 mg/kg MEQ groups. There is a downward trend in the body weight of F_1_ pups on days 1, 4, 7, 14, and 21 at 55, 110, and 275 mg/kg MEQ groups, and F_2_ pups on days 1, 4, 7, 14, and 21 at 110 and 275 mg/kg MEQ groups when compared with control group. Our findings suggested that exposure of MEQ at 110 and 275 mg/kg diets induced the developmental inhibition on pups in both generations, while the 55 mg/kg diet caused toxicity only in F1 generations. It was reported that higher dose of cyadox and quinocetone resulted in the significant decrease in the body weight of pups (Wang et al., 2011a, 2012). Here, it was presumed that the maternal toxicity that caused by MEQ at 110 and 275 mg/kg diets in F_0_ and F_1_ females resulted in worse developmental conditions in their pups.

The QdNOs derivatives, such as CBX and OLA, were reported to have reproductive and teratogenic toxicity. An aqueous solution of CBX was administered by oral gavage once daily to groups of 10 pregnant rats at a dose of 0, 10, 25, 50, and 100 mg/kg b.w. (JECFA, 2003a). Embryo lethality and teratogenicity were noted at the highest dose, the number of live fetuses being reduced to 2.4 per liter (JECFA, 2003a). The fetal sex ratio was affected at 100 mg/kg b.w./day, but not statistically significantly (JECFA, 2003a). It was concluded that maternally toxic doses of CBX caused embryo-toxicity and embryo lethality (Yoshimura, 2002). In the reproductive and teratogenic study of OLA, the rats were given oral doses of 0, 20, 60, and 180 mg/kg b.w./day as an aqueous suspension in tragacanth by gavage from day 6 to 15 of gestation (JECFA, 2003b). The results demonstrated that pregnant rats with highest daily dose showed reductions in body weights and numbers of live fetuses with a higher incidence of resorptions (JECFA, 2003b; Lorke, 1971, unpublished). OLA had a teratogenic effect at the highest dose level given to pregnant rats (180 mg/kg b.w./day) on days 6–15 of gestation (Lorke, 1971, unpublished). In the present study, the significant decreased number of total litter losses and live pups/litter (F1 and F2) generations indicated reproductive toxicity in F0 and F1 adults at 275 mg/kg MEQ group. Furthermore, the pathology findings revealed that broadening of interstitial, necrosis and dissolution of spermatogonial cells and spermatocytes in some males; the incomplete structure of the uterus and the neutrophil infiltration in submucosal glands in some females were noted in 110 and 275 mg/kg MEQ groups, suggesting that MEQ caused reproductive toxicity to Wistar rats. This finding was consistent with the previous reports revealing the testis toxicity invoked by MEQ in rats and mice (Ihsan et al., 2011; Liu et al., 2017b,c). In a previous study of acute and subchronic toxicological evaluation of MEQ in Wistar rats, MEQ at 275 mg/kg diet induced hepatic and adrenal histological changes as well as leaking of different serum constituents in Wistar rats (Ihsan et al., 2010), which is consistent with our results. In real life, the dose of animals exposed to MEQ is about 10 mg/kg body weight (Li et al., 2012), which is equivalent to 110 mg/kg MEQ diet in our study. Based on this, our study suggests that MEQ has reproductive toxicity in its clinical use. Additionally, there was significant decrease in the number of fertility index (F0 and F1), and a downward trend in fetal body weight at 110 and 275 mg/kg of MEQ groups, indicating that the certain embryo toxicity was induced by MEQ at the dose level of 110 and 275 mg/kg diets. In the teratogenicity study, significant decreases in the live birth rate, survival rate of birth and survival rate of lactation were observed in F2 generation at 55, 110, and 275 mg/kg MEQ groups, while the significant decrease in survival rate of birth was noted in F1 generation at 275 mg/kg MEQ group, indicating the stronger teratogenic to F2 generation than F1 generation. These results not only revealed that MEQ induced teratogenicity to Wistar rats, but also, indicated a certain accumulation toxicity of teratogenicity invoked by MEQ. Therefore, the teratogenicity and severe maternal toxicity induced by MEQ might be responsible for the adverse effects on maternal rats and a direct action on the conceptus, and subsequently, contributed to fetal malformations and embryo and fetal deaths.

Liver was identified as one of the main target organs for toxicity mediated by MEQ in mice (Liu et al., 2017d) and rats (Ihsan et al., 2010). ALB, ALT, ALP, and AST are general markers of liver functions, which can be observed in both cytoplasm and mitochondria of hepatocytes. MEQ induced kidney toxicity in the male Wistar rats in a sub-chronic study (Huang et al., 2010a). The changed levels of kidney indices of UA, BUN, and CREA, suggested the impairment of kidney. In the present study, the significant increases in ALT and AST were noted in 275 mg/kg F1 group; a significant reduced concentration of UA and increased level of CREA were found in in 275 mg/kg F0 group. These results indicated the chronic liver and kidney disease induced by MEQ. In the present study, the age of F0 and F1 at the time of slaughtering is different and the F0 is a little older than F1. This might be reason for the serum parameters for F0 and F1 in control group are different. Organ indices showed that the absolute and relative liver weights were changed significantly at 275 mg/kg diet in F0 females, F1 males and females. The significant change of absolute kidney weight was noted in F0 and F1 male at 275 mg/kg diet. In the histological examination, the vacuoles with a large number of blood cells, and hyperplasia of the epithelioid cells of the bile duct were noted in liver; the swelling and hyperplasia of renal vesicle wall cell, and degeneration and necrosis of renal tubular epithelium were observed in kidney at 110 and 275 mg/kg MEQ groups. Therefore, the serum biochemical results, organ indices along with the pathological changes illustrated that the liver and kidney were two main target organ for toxicity caused by MEQ. This study showed that alterations of body, organ weight with pathological change and biochemical parameters were induced by MEQ, not by abnormalities of physiological development.

The adrenal is considered as a common toxicological target for QdNOs in the endocrine system (WHO, 1991b; Huang et al., 2009). Potassium (K) is a major intracellular cation maintaining the ionic gradients for neural impulse transmission, while sodium (Na) is a major cation of extracellular fluid maintaining the water distribution and osmotic pressure. These two factors play an important role in regulating aldosterone production or release. A lot of evidences suggested that MEQ interfered the renin-angiotensin-aldosterone system with inhibition of the expression of aldosterone synthetase (Huang et al., 2009, 2010a,b; Wang et al., 2016b). CBX and OLA were also reported to interfere with biosynthesis or release of aldosterone in pigs both *in vivo* and *in vitro* (Ihsan et al., 2010). In the present study, the changed levels of Cl, K, and Na suggested that MEQ induced toxicity in salt and water balance. The absolute adrenal weight was noted in F0 female at 110 and 275 mg/kg groups, in F0 male at 55, 110, and 275 mg/kg groups, respectively. The relative adrenal weight was observed in F0 female at 275 mg/kg diet. The morphological alterations of the adrenal resulting from MEQ at 110 and 275 mg/kg groups included the proliferation of fascicular zone cell, increased binuclear cell and adrenocortical tumor. Regarding the adrenal toxicity induced by MEQ, a recent study has been conducted and the results demonstrated that the oxidative stress and metabolites of MEQ were associated with the adrenal toxicity in H295R cells that originated from a human adrenocortical carcinoma (Wang et al., 2016b). Therefore, it was suspected that the oxidative stress and metabolites of MEQ might be responsible for the changed levels of serum biochemical indices and adrenal toxicity in Wistar rats. The further study is required to illustrate the correction of these two factors in adrenal toxicity *in vivo*.

The previous studies reported that the DNA damage is firmly linked to teratogenicity (Yang et al., 2014; Si et al., 2017; Wani et al., 2017) and reproductive toxicity (Lin et al., 2014; Yang et al., 2014; Afsar et al., 2017; Adana et al., 2018). QdNOs, acting as an inhibitor of DNA synthesis, induced genotoxicity in the bacteria and mammalian cells (Cheng et al., 2015; Liu et al., 2016, 2017a; Wang et al., 2016a), suggesting that DNA damage may involve in the teratogenicity and reproductive toxicity mediated by MEQ. It is thought that the reproductive toxicants exert toxicity through oxidative damage to DNA in the germ cells and/or direct effects on the endocrine system, resulting in disruptive development and abnormalities (Doreswamy and Muralidhara, 2005; Aitken and Baker, 2006; Kumar and Muralidhara, 2007; Turner and Lysiak, 2008). As reported, the N→O group reduction was a main metabolic pathway in QdNOs metabolism (Liu et al., 2010, 2016; Liu and Sun, 2013; Cheng et al., 2015, 2016), and accompanying this, oxidative stress and drug radical intermediates had emerged that ultimately caused toxicity (Liu et al., 2017c,d). The oxidative stress and metabolites of MEQ were also considered as an important mechanism of genotoxicity (Liu et al., 2012, 2016), and organ toxicity in models of the adrenal gland (Huang et al., 2009), liver (Wang et al., 2011b; Liu et al., 2017d), spleen (Wang et al., 2011b), and endocrine and reproductive system (Ihsan et al., 2011; Liu et al., 2017b,c). Regarding the reproductive toxicity of MEQ, it was reported that the metabolites of MEQ along with the increased levels of 8-hydroxydeoxyguanosine (8-OHdG) were associated with the testis toxicity (Ihsan et al., 2011; Liu et al., 2017c). Taken together, it was suspected that oxidative stress, the metabolites of MEQ and the DNA damage may be involved in the reproductive toxicity and teratogenicity in rats invoked by MEQ, and the further study should be conducted to clarify the role of these three factors.

## Conclusion

In conclusion, the results of two generation feeding reproduction study which we described here provide a more comprehensive toxicity profile of MEQ and benefit evaluating the further risk of MEQ in food animals. MEQ induced maternal, embryo, and reproductive toxicities in rats at the doses of 110 and 275 mg/kg. MEQ depressed the development of fetus and induced teratogenicity at 55, 110, and 275 mg/kg diets. The NOAEL for reproduction toxicity of MEQ was 25 mg/kg diet. Furthermore, the present study indicted that the severe maternal toxicity of MEQ might be responsible for the adverse effects on the maternal rats, conceptus and embryo, which finally, resulting in fetal malformations and fetal deaths.

## Author Contributions

ZY, XW, and SX conceived the idea. QL and ZL analyzed and discussed the data and wrote the paper. QW, IA, MS, and ZF performed and revised the experiments. All the authors discussed the results and contributed to the final manuscript.

## Conflict of Interest Statement

The authors declare that the research was conducted in the absence of any commercial or financial relationships that could be construed as a potential conflict of interest.

## Abbreviations

ALB: albumin
ALP: alkaline phosphatase
ALT: alanine aminotransferase
ANOVA: analysis of variance
AST: aspartate aminotransferase
b.w.: body weight
BUN: blood urea nitrogen
CBX: carbadox
Cl: chloride
CREA: creatinine
DNA: deoxyribonucleic acid
GD: gestation day
H&E: hematoxylin and eosin
K: potassium
MEQ: mequindox
Na: sodium
NOAEL: no observed adverse effect level
OLA: olaquindox
PND: postnatal day
QdNOs: quinoxaline-di-*N*-oxides
TP: total protein
UA: uric acid
