# Corrigendum: The Reproductive Toxicity of Mequindox in a Two-Generation Study in Wistar Rats

**DOI:** 10.3389/fphar.2018.01489

**Published:** 2018-12-21

**Authors:** Qianying Liu, Zhixin Lei, Qin Wu, Ihsan Awais, Muhammad A. B. Shabbir, Saeed Ahmed, Zainab Fatima, Xu Wang, Yuanhu Pan, Shuyu Xie, Zonghui Yuan

**Affiliations:** ^1^National Reference Laboratory of Veterinary Drug Residues (HZAU) and MAO Key Laboratory for Detection of Veterinary Drug Residues, Huazhong Agricultural University, Wuhan, China; ^2^MOA Laboratory for Risk Assessment of Quality and Safety of Livestock and Poultry Products, Huazhong Agricultural University, Wuhan, China; ^3^Hubei Collaborative Innovation Center for Animal Nutrition and Feed Safety, Wuhan, China

**Keywords:** reproductive toxicity, teratogenicity, mequindox, Wistar rats, developmental toxicity

In the original article, there was a mistake in Figure 4 as published. Figure 4B was not displayed at the correct magnitude than that described in the figure legend. The corrected Figure 4 appears below.

**Figure 4:**
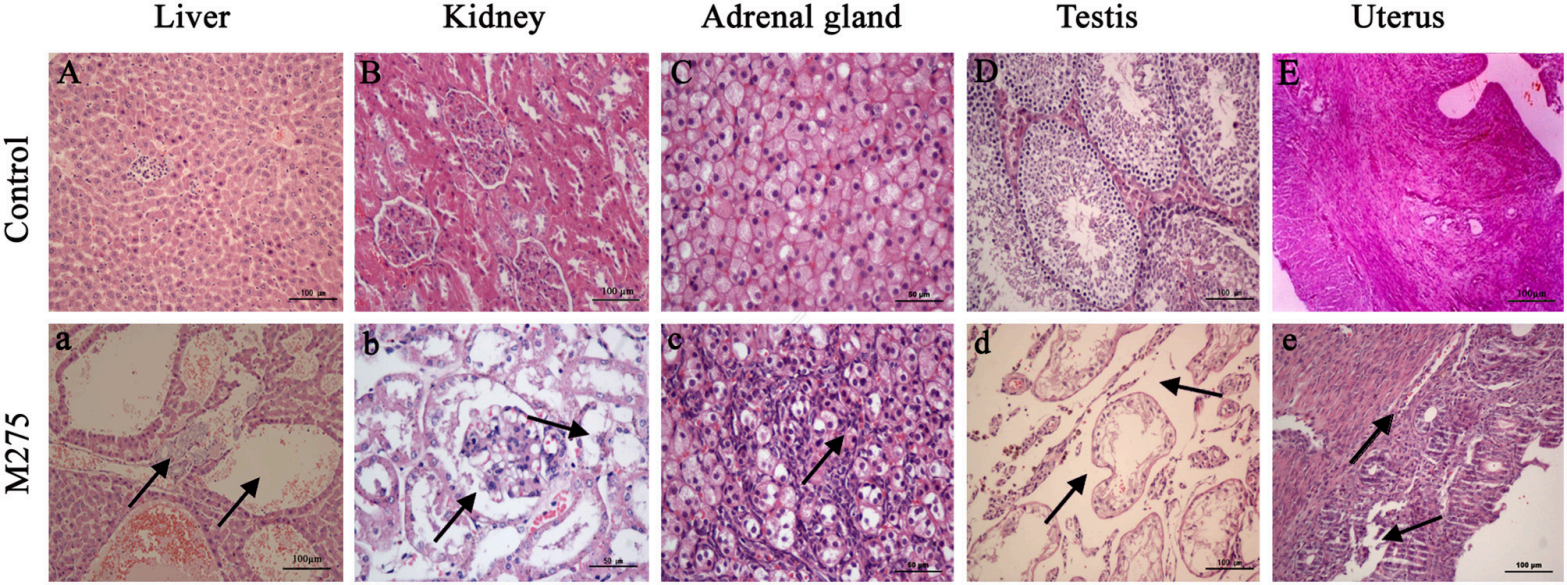
Selected microphotographs of liver, kidney, adrenal gland, testis and uterus (200X and 400X). M275, 275 mg/kg diet. **(A)** Liver (200X), **(B)** kidney (200X), **(C)** adrenal (400X), **(D)** testis (200X), and **(E)** uterus (200X) of F_0_ and F_1_ from the control group; **(a)** Liver in the 275 mg/kg MEQ group (200X). The vacuoles with a large number of blood cells, and hyperplasia of the epithelioid cells of the bile duct were marked with arrows; **(b)** Kidney in the 275 mg/kg MEQ group (400X). The swelling and hyperplasia of renal vesicle wall cell, and degeneration and necrosis of renal tubular epithelium were marked with arrows; **(c)** Adrenal gland in the 275 mg/kg MEQ group (400X). The proliferation of fascicular zone cell, increased binuclear cell and adrenocortical tumor were marked with arrows; **(d)** Testis in the 275 mg/kg MEQ group (200X). The broadening of interstitial, necrosis and dissolution of spermatogonial cells and spermatocytes in the lumen were marked with arrows; **(e)** Uterus in the 275 mg/kg MEQ group (200X). The incomplete structure and neutrophil infiltration in submucosal glands were marked with arrows.

The authors apologize for this error and state that this does not change the scientific conclusions of the article in any way. The original article has been updated.

